# Clinical management of ectopic Cushing Syndrome in neuroendocrine neoplasms: a national survey

**DOI:** 10.3389/fendo.2025.1690837

**Published:** 2025-11-07

**Authors:** Alice Laffi, Antonio Prinzi, Carla Di Dato, Tiziana Feola, Margherita Medici, Annamaria Colao, Antongiulio Faggiano, Andrea Gerardo Antonio Lania

**Affiliations:** 1 Division of Medical Oncology, Humanitas Gavazzeni, Bergamo, Italy; 2 Endocrinology Unit, Department of Clinical and Experimental Medicine, Garibaldi-Nesima Medical Center, University of Catania, Catania, Italy; 3 Department of Precision Medicine in Medical, Surgical and Critical Care (Me.Pre.C.C.), University of Palermo, Palermo, Italy; 4 Santo Spirito Hospital, Endocrinology Unit, Rome, Italy; 5 Department of Experimental Medicine, “Sapienza” University of Rome, Rome, Italy; 6 Department of Neuroendocrinology, Neuromed Institute, IRCCS, Pozzilli, Italy; 7 Department of Medical Sciences, Section of Endocrinology, Geriatrics and Internal Medicine, University of Ferrara, Ferrara, Italy; 8 Department of Clinical Medicine and Surgery, Endocrinology, Diabetology and Andrology Unit, Federico II University of Naples, Naples, Italy; 9 Endocrinology Unit, Department of Clinical and Molecular Medicine, Sant’Andrea Hospital, ENETS Center of Excellence, Sapienza University of Rome, Rome, Italy; 10 Endocrinology, Diabetology and Medical Andrology Unit, IRCCS Humanitas Research Hospital, Milan, Italy; 11 Department of Biomedical Sciences, Humanitas University, Milan, Italy

**Keywords:** ECS, EAS, Cushing Syndrome, NET, NEN

## Abstract

**Background:**

Ectopic Adrenocorticotropic Hormone (ACTH) Syndrome (EAS) is a complex disorder caused by ACTH-producing tumors located outside the pituitary gland. EAS is most commonly associated with neuroendocrine neoplasms (NENs), rare malignancies category. Due to the nonspecific symptoms, EAS is often misdiagnosed, contributing to increased morbidity and complicating clinical management. In Italy, access to diagnostic and therapeutic resources for EAS and NENs varies significantly by region. As part of the 2024–2025 NIKE (Neuroendocrine Tumors, Innovation in Knowledge and Education) initiative, a multidisciplinary group, including endocrinologists, oncologists, pathologists, and nuclear medicine experts, designed a national survey to assess awareness, diagnostic approaches, and management of EAS in Italian centers.

**Methods:**

A 50-items structured questionnaire was developed, covering 3 sections: respondents’ profile, diagnostic approaches, and treatment strategies. The survey was distributed as an anonymized form via email, with data collected from April to June 2025.

**Results:**

Sixteen Italian centers with NEN and EAS expertise participated. Most experts worked in European referral centers for rare tumors where the majority have an in-house, NEN-dedicated multidisciplinary team. Initial points of contact occurred most frequently in oncology (37.5%) and endocrinology (31.5%) clinics. A diagnostic delay was reported by 56% of respondent centers; hypokalemia was the most common presenting sign (93.8%). In 56.3% of centers, respondents reported that EAS was more commonly diagnosed before the detection of the underlying NEN, most frequently lung carcinoids or small/large cell cancers (87.5%). Regarding diagnostic practices, 56.3% of centers indicated the use of the 1 mg dexamethasone suppression test (DST), followed by the high-dose DST. The desmopressin test was considered outdated or replaceable by 43.8% of respondents. Regarding therapeutic approaches, respondents reported that upfront surgery was performed in up to 50% of centers, with preoperative bridging pharmacological therapy used to achieve eucortisolism. Osilodrostat was the most frequently preferred first-line treatment.

**Conclusion:**

This survey provides a valuable snapshot of EAS care in Italy, highlighting both strengths and areas for improvement. The findings underscore the need for a national, more structured referral network to ensure timely diagnosis and access to specialized care. These insights may guide national protocol harmonization in EAS management and better alignment with international standards.

## 1 Introduction

Ectopic Adrenocorticotropic Hormone (ACTH) Syndrome (EAS) is a complex pathological condition caused by ACTH-producing tumors located outside the pituitary gland (1). EAS is most commonly linked to neuroendocrine neoplasms (NENs), a rare and heterogeneous group of tumors capable of originating in almost any organ system (2–4). Cushing’s syndrome (CS) itself is uncommon, with an estimated annual incidence of 2–3 cases per million individuals (5). Approximately 80–85% of CS cases are ACTH-dependent and 15–20% are ACTH-independent; among the ACTH-dependent forms, about 20% are due to EAS (6). Among NENs, the most frequent sources of ectopic ACTH production are lung carcinoids (>25%), followed by thymic carcinoids (TC, 11–25%, including cases associated with Multiple Endocrine Neoplasia type 1, MEN-1), pancreatic neuroendocrine tumors (8%), medullary thyroid carcinomas (MTC, 6%), and pheochromocytomas (5%) (7–9). EAS presents with a wide spectrum of clinical manifestations, often influenced by the nature and location of the underlying tumor and the degree of hypercortisolism. The symptoms can range from mild to severe, and in some cases, the condition may initially be misdiagnosed due to its nonspecific presentation. Common features include hypertension, diabetes mellitus, muscle weakness, and metabolic alkalosis, while skin changes (e.g., bruising, striae, hyperpigmentation) are frequent but less specific (10, 11). Psychiatric symptoms, such as anxiety, depression, and cognitive impairment, are common and can sometimes lead patients to seek care from neuropsychiatrists rather than endocrinologists. Moreover, systemic, local, or multiple infections, as well as osteoporosis, osteopenia, and fractures, have been reported and correlate with the degree of hypercortisolemia (1, 12). Diagnostic delay is a significant clinical issue in EAS: symptoms of hypercortisolism are often nonspecific or overshadowed by those of the primary tumor, leading to delayed recognition. A recent meta-analysis reported an average diagnostic delay of approximately 14 months, although patients with EAS typically have a shorter time to diagnosis compared with those with adrenal or pituitary CS (13). Nevertheless, these delays contribute to increased morbidity and complicate clinical management, highlighting the need for heightened awareness among clinicians. According to the most recent international consensus on Cushing’s syndrome (14), no single biochemical test can reliably distinguish between pituitary and ectopic ACTH secretion. The diagnostic approach should combine clinical assessment, hormonal testing, and imaging. A noninvasive combination of high-dose dexamethasone suppression and CRH or desmopressin stimulation tests, together with pituitary Magnetic Resonance Imaging (MRI), may help differentiate the source of ACTH excess. When results are discordant or MRI findings are negative or inconclusive, bilateral inferior petrosal sinus sampling (BIPSS) remains the gold standard. In patients with clinical features suggestive of ectopic ACTH secretion, a thin-slice Computed Tomography (CT) scan from neck to pelvis is recommended to identify potential neuroendocrine tumors.

In Italy, access to diagnostic and therapeutic resources for EAS and NENs varies significantly by region. Currently, eight European Neuroendocrine Tumor Society (ENETS) centers of excellence are accredited: five located in northern Italy, two in Rome, and one in Naples. As result, central and southern Italy, including the islands remain relatively underserved. As part of the 2024–2025 NIKE (Neuroendocrine Tumors, Innovation in Knowledge and Education) program, a multidisciplinary group, including endocrinologists, oncologists, pathologists and nuclear medicine specialists with a special interest in neuroendocrine issues, collaborated to identify key questions needed to advance the understanding of EAS and NENs. The present study was therefore designed to conduct a nationwide survey evaluating awareness, diagnostic strategies, and therapeutic approaches to EAS in Italian clinical centers.

## 2 Methods

The topic of the survey was identified during the 2024 annual NIKE meeting, coordinated by AGL. A structured questionnaire comprising 50 items was developed and organized into three thematic sections (see **Supplementary Material S1**). Questions (Q)1–19 explored the general profile of the respondent, including medical specialty, geographic location, and experience with NENs/EAS (prepared by AL), Q20-Q31 addressed diagnostic approaches to EAS and NEN (prepared by CDD, AP), covering access to imaging, biochemical tests, and the availability of multidisciplinary teams, and Q32-Q50 focused on EAS treatment strategies, including pharmacological therapies, surgical interventions, and follow-up protocols (prepared by TF, MM).

Within the first section (Q1-Q19), questions Q1-Q3 referred to the professional background of respondents, Q4-Q10 to the characteristics of the local healthcare setting, Q11-Q19 to the hospital experience of the specialist. The initial draft of the questionnaire was prepared by AGL and AL, and subsequently reviewed and validated by the coordinators of the 2024 NIKE group. The validating panel included endocrinologists, oncologists, nuclear medicine specialists, and pathologists, with additional input from Oncological Endocrinology (ENDO ONCO) club of the Italian Endocrine Society (SIE). A validation step was conducted to ensure relevance, clarity, and comprehensiveness of the items.

### 2.1 Survey participants

NEN specialists affiliated with hospitals and research institutes across Italy were identified from the ENDO-ONCO Club of SIE and the NIKE study group membership lists. All centers with at least one clinician experienced in managing NEN/EAS were invited to participate. From each center, a single response was requested, preferentially from the specialist with the most experience in NEN/EAS management, typically an endocrinologist or oncologist. If multiple eligible specialists were present at a center, the center itself determined who would respond. The survey was distributed as an anonymized online form via email, and data were collected between April and June 2025. Particular attention was paid to achieving adequate geographic representation, considering the uneven distribution of ENETS Centers of Excellence in Italy.

### 2.2 Data analysis

Descriptive statistics were used to summarize respondent demographics, institutional characteristics, and reported diagnostic and treatment practices. Comparative analyses were conducted to explore regional disparities in EAS management and resource availability. Agreement among the responders was considered for at least a 75% of concordance.

## 3 Results

Answers from a total of 16 out 26 centers with expertise in NEN and EAS fields working in hospitals throughout Italy have been received and collected. Q1-Q18 collected details about general features of the respondents. **Table 1** summarizes these characteristics.

**Table 1:** Features of responders.

	n (%)
Geographical location of Italy	
North	7 (44)
Center	2 (12.5)
South	4 (25)
Islands	3 (19)
Age	
< 50 years	4 (25)
≥ 50 years	12 (75)
Gender	
Female	6 (37.5)
Male	10 (62.5)
Other	0
Center of excellence	
ERN	4 (25)
ENETS	5 (31)
EURACAN	3 (19)
None	7 (44)
Unknown	0
Guidelines employed	
ENETS	16 (100)
ESMO	5 (31)
NCCN	3 (19)
AIOM/ITANET	9 (56)
NANETS *more than one guideline can be consulted	2 (12.5)
Availability of a NEN-dedicated multidisciplinary team in the hospital	
Yes	14 (87.5)
No	2 (12.5)
NEN patients N°/year per hospital	
< 50	3 (19)
50 - 100	6 (37.5)
100 - 200	3 (19)
> 200	4 (25)
Cushing Syndrome cases N°/year per hospital	
1 - 2	1 (6)
3 - 6	2 (12.5)
7 - 10	5 (31)
> 10	8 (50)
EAS cases N°/yeas per hospital	
1 - 2	11 (70)
3 - 6	4 (25)
7 - 10	1 (6)
EAS patient access	
Through inpatient consultation	8 (50)
Referred by clinical/surgery outpatient clinics	3 (19)
Referred by family doctor	2 (12.5)
Referred by another hospital	2 (12.5)
Self-booking by the patient	1 (6)

ERN, European Reference Networks; ENETS, European Neuroendocrine Tumors Society; EUROCAN, Expert Care for Rare Adult Solid Cancers; ESMO, European Society of Medical Oncology; NCCN, National Comprehensive Cancer Network; AIOM/ITANET, Associazione Italiana di Oncologia Medica/Italian Association for Neuroendocrine Tumours; NANETS, North American Neuroendocrine Tumor Society; NEN, neuroendocrine neoplasm; EAS, Ectopic ACTH Syndrome.

**Figure 1:**
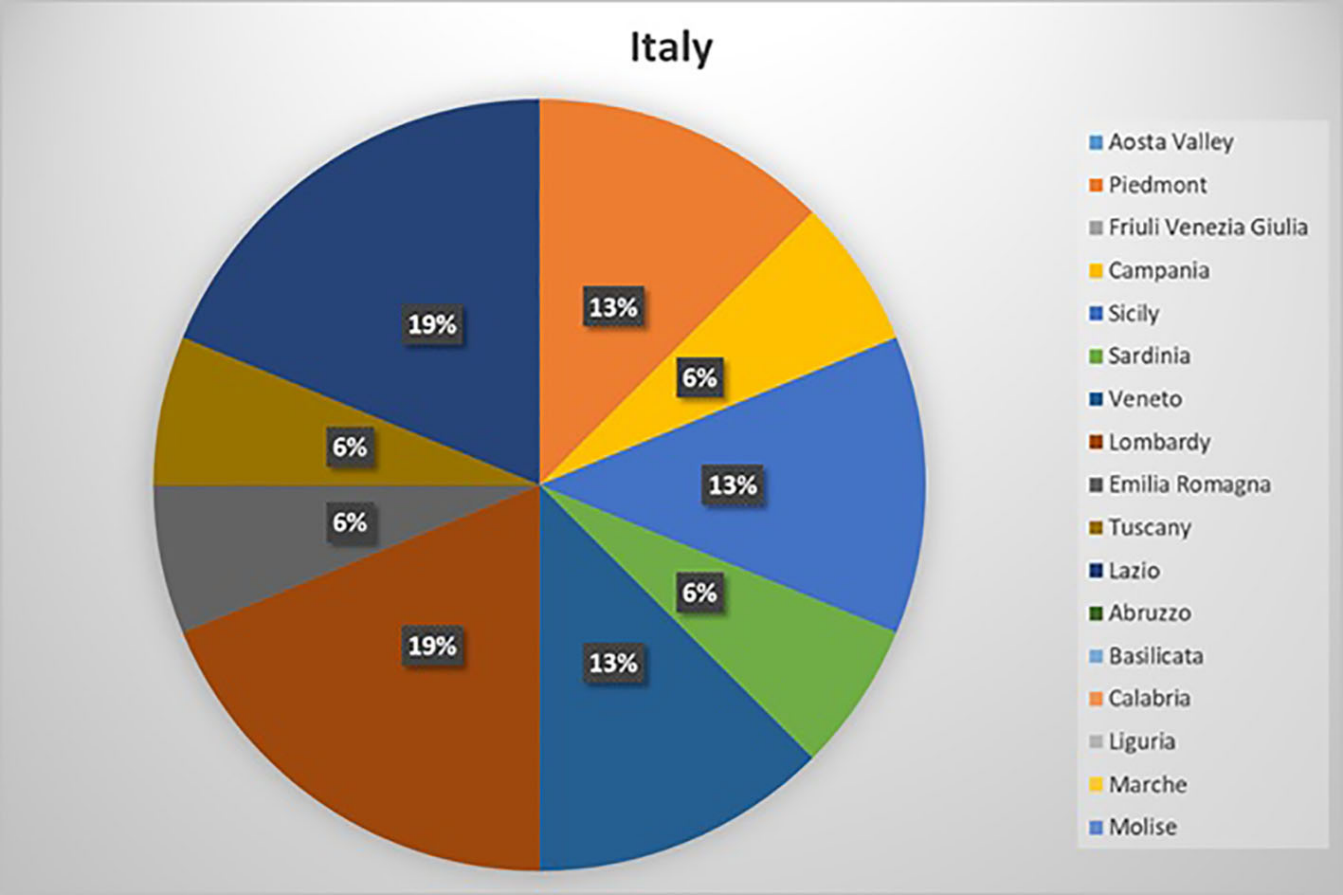
Centers answers by geographic areas. The distribution of the response across Italy, by different geographic areas.

We received at least one answer from every geographic area (**Figure 1**). Most of the involved experts worked in a European referral center for rare tumors including centers of excellence endorsed by ENETS, ERN (European Reference Networks) and/or EUROCAN (Expert Care for Rare Adult Solid Cancers). Some centers met the eligibility criteria to participate in all the networks based on their qualifications and specialization areas. Among those centers not officially part of the European networks, only one center usually refers patients to another Center of Excellence. Among the participating centers, 13 out of 16 (81.5%) reported managing more than 50 patients with NEN annually, and 7 out of 16 (44%) managed over 100 patients per year. Additionally, 13 out of 16 centers (81.5%) of centers manage more than 7 cases of CS annually, of which approximately 70% were attributed to EAS.

Most centers have a NEN-dedicated multidisciplinary team within the hospital. In centers without an in-house team, patients are referred to a multidisciplinary board at another institution, primarily to ensure access to specialized personnel and advanced technologies, such as specific imaging modalities or laboratory tests, required for optimal management of patients with NEN. Regarding patient access pathways, the most frequent initial points of contact are oncology (37.5%) and endocrinology (31.5%) outpatient clinics followed by surgery and gastroenterology outpatient clinics, as well as day hospitals multidisciplinary consultations for patients referred from other hospitals.

Regional differences were observed in referral patterns: in Northern Italy, patients with NEN are more frequently referred to oncologists or surgeons (5/8 answers), whereas in Southern regions, referrals are more commonly directed to endocrinologists (4/5 answers).

### 3.1 Personal experience

Q 17–19 explored the specialists’ experiences during the early stages of the diagnosis of EAS and NEN. Most centers reported a delay between the onset of symptoms or biochemical abnormalities and the final diagnosis, often associated with inappropriate treatments due to misdiagnosis of the endocrine disorder or following the initial NEN diagnosis (reported by 56% of respondents). Regarding the sequence of diagnoses, 56.3% of centers indicated that the diagnosis of EAS preceded the identification of the NEN, while in approximately 31% of centers the NEN diagnosis preceded EAS. In the remaining cases, both conditions were diagnosed simultaneously.

EAS was most frequently associated with lung carcinoids or small/large cell pulmonary cancer as reported by 87.5% of centers. The remaining centers reported a link between EAS and pancreatic NENs. No cases of EAS linked to pheochromocytoma, TC or MTC.

### 3.2 Diagnostic approach

Q 20–31 explored the diagnostic approach to EAS and NENs across different geographic regions, focusing on commonly adopted diagnostic algorithms in suspected cases of EAS.

Among respondents, 56.3% preferred to start with the 1 mg dexamethasone suppression test (DST), followed by the high-dose dexamethasone suppression test (HDDST). Conversely, 37.5% preferred starting with the HDDST, followed by the corticotropin-releasing hormone (CRH) test. A minority (6.3%) reported beginning with the 1 mg DST followed by the desmopressin (DDAVP) test. HDDST and CRH tests seem to be preferred by experts from Norther Italy, while the sequence 1 mg DST followed by HDDST seems to be uniformly adopted across the country.

Q 21 aimed to identify which of the listed tests are considered outdated or potentially replaceable. Most respondents (43.8%) indicated the DDAVP test, followed by low-dose DST, BIPPS and CRH test. The omission of BIPSS in the diagnostic phase was reported solely by respondents from Northern Italy. While 75% of participants reported having access to all required diagnostic laboratory tests, the remaining 25% were equally divided between those lacking access to BIPSS and those lacking access to the CRH test, despite it being considered essential. Notably, none of the respondents regarded the DDAVP test as essential enough to be acquired if unavailable at their institution. For imaging in suspected EAS associated with a NEN, 75% of centers reported using CT as the first-line modality, with MRI as an alternative. Functional imaging, such as ^68^Gallium (^68^Ga) DOTA-peptide positron emission tomography (PET)/CT or PET-DOPA, was selected based on clinical features. None of the respondents reported using 18F-Fluorodeoxyglucose (FDG) PET/CT or Indium-111 pentetreotide (Octreoscan^®^) as initial imaging. These imaging modalities were generally available either within the centers or at a regional level. As second-line images, 81.3% of respondents reported ^68^Ga DOTA PET/CT or PET-DOPA, while a small percentage used ^18^FDG PET or additional morphological imaging.

From a clinical perspective, hypokalemia was identified as the most frequent initial sign of EAS by 93.8% of respondents. Only 18.7% NEN associated with EAS. Q 29–30 explored potential diagnostic delays. A total of 75.1% of respondents reported a delay of 1–3 months between the start of diagnostic workup and the first specialist evaluation with no significant differences among the regions. However, 81.3% indicated that an EAS diagnosis was typically achieved within one month of patient referral to an expert endocrinologist.

### 3.3 Therapeutic approaches

**Figure 2:**
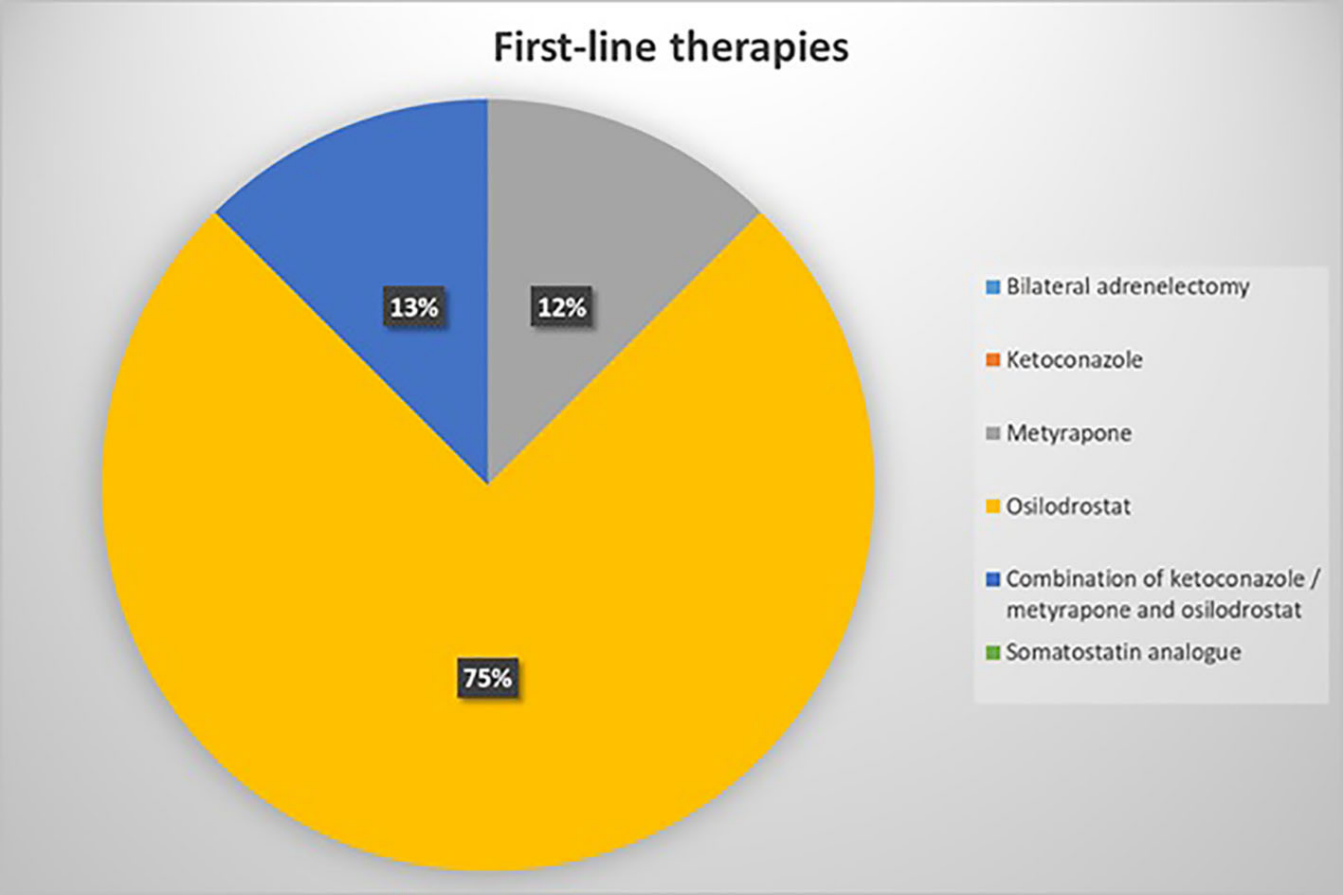
First-line systemic treatments for Ectopic ACTH Syndrome (EAS) management. Osilodrostat, metyrapone or the combination of ketoconazole, metyrapone and osilodrostat were the most common first-line therapies.

According to survey responses, the management of patients with EAS in Italy appears to be evenly distributed across inpatient services, outpatient clinics, and day hospital settings. Upfront surgical intervention was recommended by approximately 50% of respondents as the preferred initial strategy. In this context, all centers consistently reported the use of bridging pharmacological therapy to achieve eucortisolism prior to surgery. The main reasons cited for excluding upfront surgery were the presence of unresectable metastatic NENs (62.5% of responders) or the occult localization of the primary tumor at the time of evaluation (37.5%). Regarding first line systemic therapies, osilodrostat was the preferred option among most respondents, primarily based on personal clinical experience. In contrast, only 30.8% reported that their treatment choices were predominantly guided by established clinical guidelines (**Figure 2**).

**Figure 3:**
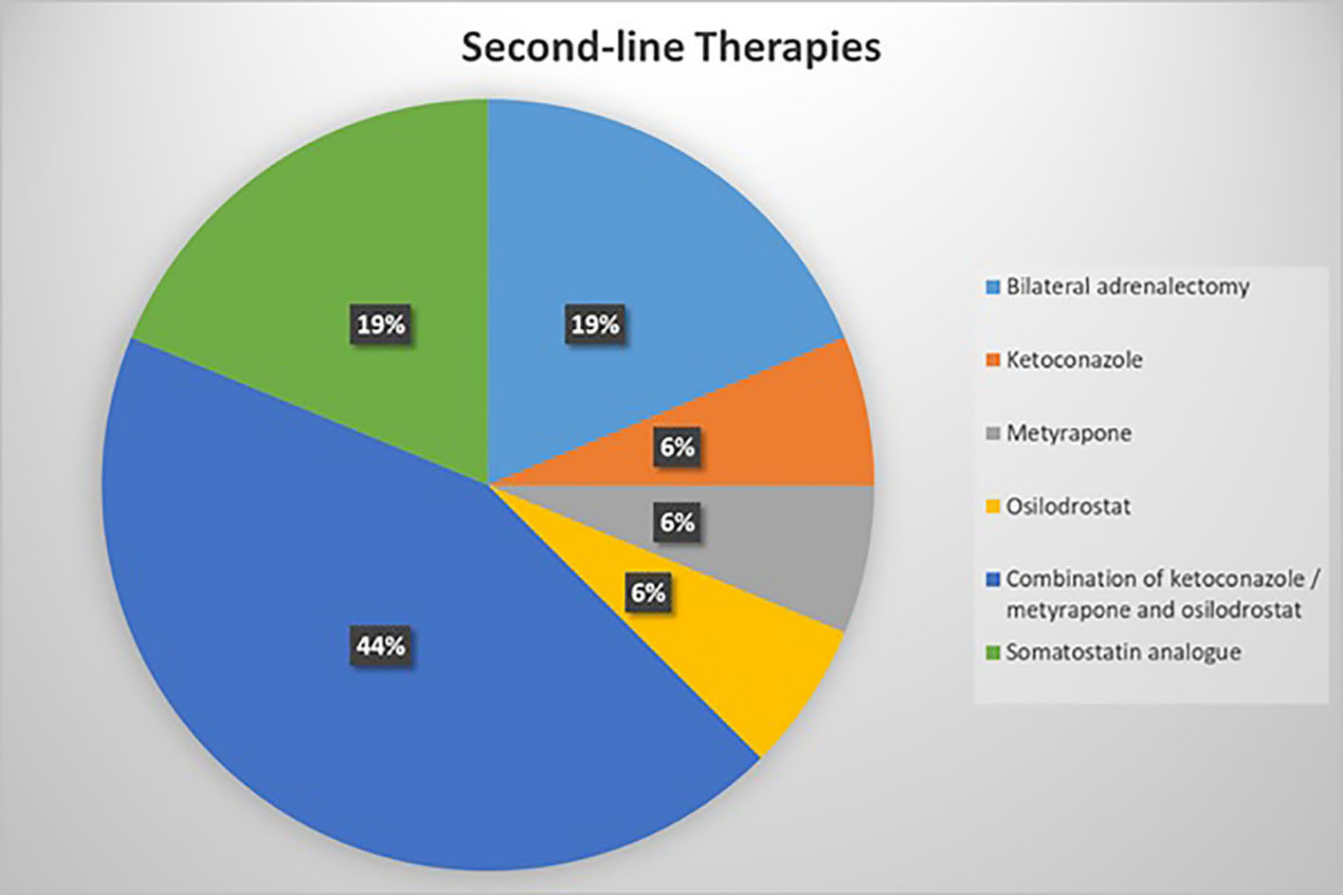
Second-line systemic treatments for EAS management. The combination of ketoconazole, metyrapone and osilodrostat were the most common first-line therapies, followed by bilateral adrenalectomy and somatostatin analogue therapy.

Responses concerning second-line treatment options were more heterogeneous. The largest proportion of respondents (43.8%) reported favoring combination therapies with two or more steroidogenesis inhibitors (SIs), typically ketoconazole, metyrapone, and/or osilodrostat. This was followed by the use of a first-generation somatostatin analogue (18.8%) or bilateral adrenalectomy (18.8%) as second-line strategies. A smaller proportion of respondents reported employing SI monotherapy (ketoconazole, osilodrostat, or metyrapone) as their preferred approach (**Figure 3**).

The decision to initiate second-line treatment was primarily reported to be driven by clinical progression of the syndrome (75%). Other influencing factors included treatment-related adverse events, drug–drug interactions with therapies for the underlying NEN, or a preference for using multiple SIs in combination. Notably, none of the respondents cited regional availability or logistical constraints as reasons for not accessing their preferred first-line agents. Q 44–50 focused on the personal therapeutic strategy and the experience of the centers in managing treatment-related toxicity. In the context of first-line treatment with SIs, 43.8% of respondents indicated adopting a dose-titration approach, while 25% reported using a block-and-replace regimen. An additional 31.3% reported reserving the block-and-replace approach for patients with aggressive disease (e.g., rapidly worsening symptoms, severe hypercortisolism, or biochemical instability requiring hospitalization).

Hepatotoxicity was identified as the most frequent adverse event leading to SI discontinuation (50% of responders), followed by gastrointestinal intolerance (18.8%). However, 25% of respondents indicated that they had not encountered adverse events necessitating treatment discontinuation.

Q 47–49 explored the use of mass spectrometry (MS) for the measurement of 24-hour urinary free cortisol during the follow-up of medical therapy with osilodrostat or metyrapone. In most centers, MS results were available within 14 days, although in 33.3% of centers turnaround time exceeded 14 days. Overall, 43.8% of respondents reported using MS in their own centers, 12.5% did not have local access but sent samples to external laboratories, and the remaining 43.8% did not use the method at all.

Finally, respondents were asked to estimate the proportion of their patients achieving biochemical and clinical control of EAS with available therapies. The majority (50.0%) reported achieving control in 50–80% of patients, while 31.3% of reported control in 80–100% of patients. In contrast, 12.5% estimated control in only 20–50% of cases, and 6.2% in fewer than 20%.

## 4 Discussion

EAS remains one of the most challenging forms of hypercortisolism to diagnose and manage, owing to its rarity, heterogeneous clinical presentation, and the variable behavior of the underlying NENs. Despite its severity and associated morbidity, few studies have systematically assessed real-world clinical practices in its management. This nationwide survey provides the first structured overview of current practices in the diagnosis and management of EAS across italian clinical centers, with a particular focus on centers managing NENs. The results highlight several important findings regarding awareness, diagnostic strategies, therapeutic approaches, and regional disparities in access to specialized resources. The survey highlights at least three key insights into the real-world management of EAS and NENs in Italy.

First, while the initial selection of centers through the ENDO−ONCO and NIKE networks may have influenced the representation of participating italian centers, the limited number of responses from certain geographic areas might also reflect the uneven distribution of specialized centers for EAS and NETs across the country. This variation may suggest regional disparities in access to care for patients residing in those areas. In regions where specialized facilities are less densely distributed, patients may face the consequential logistical and psychological challenge associated with traveling longer distances to receive appropriate care. Moreover, the observed differences in patients access modalities and diagnostic sequencing across Italy likely reflects disparities in local healthcare infrastructure and resource allocation, which may, in turn, influence both the timing and diagnostic pathway.

These findings emphasize the urgent need to establish a coordinated hub-and-spoke network, ensuring more equitable access to expert management across all geographic areas.

Second, diagnostic delay in patients with EAS is a well-recognized challenge. As anticipated earlier, literature data show that the mean time from symptom onset to diagnosis in EAS is approximately 14 months (13). However, our survey indicates that most italian centers achieve diagnosis within 1–3 months of referral to expert care and no significant regional differences in diagnostic delay were observed, suggesting that specialized expertise and structured pathways can markedly reduce delays. As previously mentioned, our survey was conducted among centers specialized in EAS and NEN fields, characterized by high levels of clinical expertise and the implementation of standardized diagnostic and therapeutic protocols. While this setting may limit direct comparability with broader international data, it underscores the availability of coordinated, specialized care in Italian centers.

Nonetheless, the interval between initiation of the diagnostic process and specialist evaluation at a referral center tends to be longer in Northern Italy, albeit without reaching statistical significance. This may, in part, be attributable to the higher density of tertiary care hospitals in the North, where the widespread availability of advanced diagnostic tools may reduce the perceived need for early referral to dedicated expert centers. Additionally, in Northern Italy, patients with EAS and NENs are often initially referred to oncologists or surgeons who, despite their expertise in managing NENs, may have limited awareness of the specific challenges associated with EAS, potentially contributing to delays in appropriate specialist evaluation.

Third, concerning the diagnostic tools for EAS, the DDAVP test was consistently reported across italian centers as having limited clinical utility. This perception is in line with the growing body of evidence highlighting the test’s limited specificity in differentiating Cushing’s disease from EAS and non-neoplastic hypercortisolism (NNH) states.

Several studies have highlighted the potential for diagnostic overlap, with DDAVP test occasionally eliciting a positive response in patients with EAS, thus increasing the risk of misclassification as pituitary-dependent CS (15). These findings are consistent with the results of a recent review by Pinelli et al., which reported a variable specificity for DDAVP test ranging from 40% to 81% in the differential diagnosis (16). Moreover, a recent meta-analysis further confirmed that the specificity of the DDAVP test is lower than that of other dynamic tests, such as CRH stimulation or the high-dose DST (17).

A more conservative approach to BIPSS was noted in Northern Italy. This aligns with the ongoing debate in the literature regarding its indications. While several authors emphasize the crucial role of BIPSS, particularly in differentiating between pituitary CS and EAS (18), underlying the low rate of neurological complication (19, 20), while others (21) highlight that, although rare, complications may still occur. Current consensus suggests that BIPSS should be reserved for selected cases of diagnostic uncertainty and performed exclusively in centers with appropriate expertise and neuro-interventional support. According to the most recent international consensus (14), a noninvasive alternative combining HDDST and CRH or DDAVP tests, together with pituitary MRI, can predict Cushing’s disease; when results are discordant, BIPSS remains the diagnostic gold standard. However, recent systematic reviews and meta-analyses have highlighted that second-line dynamic testing, particularly CRH stimulation, shows high diagnostic accuracy for distinguishing Cushing’s disease from ectopic ACTH secretion, especially when availability and cost limit BIPSS use (17). In selected patients, the combined use of negative pituitary MRI, negative CRH/DDAVP tests, and positive whole-body CT findings may even allow clinicians to bypass BIPSS when evaluated by an experienced multidisciplinary team (22).

Interestingly, ¹^8^FDG PET/CT was not reported among the most frequently used imaging modalities. This likely reflects its selective indication in cases of high-grade NENs, whereas somatostatin receptor-based PET tracers (e.g., ^68^Ga-DOTA-PET/CT) remain the preferred functional imaging tools in the initial diagnostic work-up of EAS. This pattern may also mirror the patient mix in the participating centers, which was likely composed mainly of well-differentiated rather than poorly differentiated NENs.

Fourth, with the therapeutic approach to EAS, international guidelines on the management of hypercortisolism recommend the use of SIs through different therapeutic strategies, tailored to the severity of the disease. According to our survey, these recommendations are consistently implemented in clinical practice across Italy (23, 24). Notably, 5 out of 16 centers explicitly reported using a block-and-replace strategy in cases of severe or aggressive cases, in alignment with the Endocrine Society guidelines, which recommend a dose titration approach for patients with milder forms of hypercortisolism.

Among the available therapies, osilodrostat was the most frequently selected first-line SI. Its use, however, appears to be primarily driven by clinical experience rather than formal guideline recommendations. This approach is appropriate, as the current Endocrine Society guidelines do not yet include osilodrostat, and its approval for the treatment of hypercortisolism, including CS and EAS, is supported primarily by emerging evidence from case reports and small series (25–29). These findings reflect a broader trend in clinical endocrinology, where real-world experience often precedes formal recommendations, especially in rare and challenging conditions like EAS.

This study presents several strengths and limitations that warrant discussion. Of the 26 centers identified within the ENDO-ONCO and NIKE networks, responses were obtained from 16. While this includes a substantial proportion of expert institutions, the limited participation, in part due to the relatively short timeframe for data collection, constrains the ability to capture a complete national picture. In particular, regions in Eastern and parts of Southern Italy were underrepresented, with most responses originating from Northern centers. A further limitation lies in the targeted inclusion of only centers with established expertise in EAS and/or NENs. Although this approach ensured high-quality responses, it excluded non-specialized centers, which are essential for understanding referral pathways and potential delays in diagnosis at the general hospital level. Finally, we also acknowledge that the survey design did not allow a detailed assessment of the sequence and individual selection of diagnostic tests for EAS. This choice was intentional to maintain conciseness and ensure completion, but it may have limited the granularity of the data. Similar variability in diagnostic approaches has also been described in the ERCUSYN survey (30).

Nonetheless, the study has key strengths. Involving expert centers allowed for a detailed assessment of current practices in managing EAS in the context of NENs. These institutions are among the most experienced in Italy and reflect high standards in diagnostics and treatment. While not all participating centers were formally affiliated with networks like EURACAN or ENETS, most were high-volume institutions managing over seven Cushing Syndrome cases annually. Their geographic distribution supports a reasonable national representation of specialized care. Finally, nearly all centers reported having a dedicated NEN multidisciplinary team; those lacking such a structure maintain active collaboration with external boards. This confirms the integration of multidisciplinary approaches in the management of complex endocrine disorders such as EAS.

## 5 Conclusion

The present survey provides a valuable snapshot of the current landscape of EAS management in Italy, highlighting both strengths and areas for improvement. The findings underscore the importance of a more structured, nationwide referral network to ensure timely diagnosis and equitable access to specialized care. Furthermore, the survey confirms key trends in diagnostic and therapeutic approaches, including the limited utility of the DDAVP test, the nuanced role of BIPSS, and the increasing reliance on SIs, particularly osilodrostat, as part of a tailored treatment strategy.

Overall, these insights may inform future efforts to optimize the management of EAS within care pathways for NENs and support the development of harmonized national protocols aligned with international guidelines.

## Data Availability

The datasets presented in this study can be found in online repositories. The names of the repository/repositories and accession number(s) can be found in the article/**Supplementary Material**.
