# Supplementary material for: Clinical management of ectopic Cushing Syndrome in neuroendocrine neoplasms: a national survey
Source: Front Endocrinol (Lausanne). 2025 Nov 7;16:1690837. doi: 10.3389/fendo.2025.1690837 (PMC12634317; doi:10.3389/fendo.2025.1690837)
Supplement: Supplementary file 1 [file DataSheet1.docx]

Supplementary Material

**S1 Supplemental: Survey’s questions**

| Q1 Italian region where you work |
| --- |
| Q2 How long has it been since you finished your training? |
| Q3 Which gender do you identify with?  Female  Male  Other  I prefer not to answer |
| Q4 At the present, do you work at a referral Center for Neuroendocrine Neoplasms (NENs) (such as ERN, EURACAN, ENETS)?  Yes , ERN  Yes, ENETS  Yes , EURACAN  No  I don’t’ know |
| Q5 If no, do you generally refer NEN patients to other hospital or research institute? |
| Q6 Which international guideline to you consider for NENs management?  ENETS  ESMO  NCCN  AIOM/ITANET  NANETS |
| Q7 Do you have an in-house NEN-dedicated multidisciplinary board? |
| Q8 If no, do you refer NEN patients to other hospitals or research institutes with a NEN-dedicated multidisciplinary board? |
| Q9 In your hospital, do you have all the specialists and facilities necessary for an adequate diagnosis and staging of neuroendocrine tumors (NETs) (radiologists, pathologists, nuclear medicine specialists, surgeons, oncologists, gastroenterologists, endocrinologists)? |
| Q 10 If not, are you able to refer NEN patients to a regional center that provides access to all the necessary specialties and diagnostic facilities not available at your institution? |
| Q 11 In your hospital what is the primary entry point for NEN patients?  Oncologic outpatient clinic  Endocrinology outpatient clinic  Surgical outpatient clinic  Gastroenterology outpatient clinic  Day Service  Tumor board evaluation of the case referred by other institutes |
| Q12 The number of NEN patients managed at your hospital per year |
| Q13 The number of patients with Cushing's syndrome managed at your hospital per year |
| Q14 How many of these are ectopic Cushing's syndrome cases? |
| Q15 How is the patient with suspected ectopic Cushing’s syndrome typically referred to the endocrinology outpatient clinic?  Referral from the general practitioner  Referral/consultation requested by clinical department  Self-referral by the patient  Referral from clinical/surgical outpatient clinic of the same hospital  Referral from another hospital |
| Q16 In the majority of ectopic Cushing's syndrome cases you have managed, the diagnosis:  was made easily and quickly from the onset of biochemical alteration or signs and symptoms  was delayed relative to the onset of biochemical changes or signs and symptoms, leading to incorrect treatments caused by a misdiagnosis of the endocrinological condition.  was made retrospectively, after the identification of the NET |
| Q 17: If other, please specify |
| Q 18 In clinical practice, in cases of ectopic Cushing's syndrome, which of the following situations occurs most frequently  the diagnosis of NET more likely precedes the endocrinological evaluation and the subsequent diagnosis of ectopic Cushing's syndrome  The diagnosis of ectopic Cushing's syndrome is typically established first, followed by subsequent identification of the underlying NEN  The diagnosis is often made concurrently with the identification of the NEN |
| Q 19 In your personal experience, which NEN is most commonly diagnosed in association with ectopic Cushing's syndrome?  Pulmonary carcinoids / small cell / large cell lung carcinomas  Neuroendocrine tumors of the pancreas  Pheochromocytoma  Thymic carcinoid  Medullary thyroid carcinoma |
| Q 20 What is the most appropriate diagnostic pathway in the evaluation of ectopic Cushing's syndrome?  1 mg dexamethasone suppression test (DST), followed by high dose DST (HDDST)  HDDST followed corticotropin-releasing hormone (CRH) test  1 mg DST followed by desmopressin (DDAVP) test  1 mg DST followed by bilateral inferior petrosal sinus sampling (BIPSS) |
| Q 21 Which of the following tests for the diagnosis of Ectopic Cushing's do you consider negligible or replaceable with another?  CRH test  DDAVP test  BISPP  1 mg DST |
| Q 22 Which imaging do you most frequently use as a first-line approach for the diagnosis of NETs associated with Ectopic Cushing' syndrome? |
| Q 23 If other, please specify |
| Q 24 In clinical practice, are all the listed tests or imaging ones that you routinely use?  No, because they are not available in my hospital/in my geographic area  No, but I can refer the patient to a hospital in my geographic area for specific tests  Yes, I can proceed with any of the abovementioned tests |
| Q 25 Which imaging do you consider crucial for the ectopic Cushing' syndrome diagnosis but is not available in your hospital or local geographic area? |
| Q 26 Which second-line diagnostic procedure do you typically use to complete the diagnosis and staging in case of a suspected NET linked to Ectopic Cushing's syndrome? |
| Q 27 Which laboratory test do you consider crucial for the ectopic Cushing' syndrome diagnosis but is not available in your hospital or local geographic area? |
| Q 28 What clinical or biochemical sign most commonly prompts referral of a patient with Ectopic Cushing's Syndrome to your attention? |
| Q 29 How long, on average, does it take from the initial diagnostic evaluation to the patient being referred to you? |
| Q 30 What is the average time between the patient’s referral to you and the diagnosis of Ectopic Cushing's Syndrome? |
| Q 31 How often do you diagnose Ectopic Cushing's Syndrome from an unknown primary tumor? |
| Q 32 Where the patient with suspected ectopic Cushing is managed?  Inpatient setting  Outpatient setting  In Day Hospital / Day Service  Other |
| Q 33 If other, please specify |
| Q 34 How many patients with ectopic Cushing do you treat with an upfront surgical approach to remove the ACTH-secreting lesion as the primary treatment? |
| Q 35 When surgery is indicated, do you start bridging medical therapy to achieve eucortisolism prior to the procedure? |
| Q 36 What is the primary reason for not proceeding with upfront surgery?  Unresectable metastatic disease  ACTH-secreting unknown primary  Patient unfit for surgery due to complications of ectopic Cushing’s syndrome  Other |
| Q 37 If other, please specify |
| Q 38 Which of the following strategies is proposed at your center as first-line treatment for the control of ectopic Cushing’s syndrome?  Bilateral adrenalectomy  Ketoconazole  Metyrapone  Osilodrostat  Combination of ketoconazole/metyrapone and osilodrostat  Somatostatin analogue |
| Q 39 Can you justify your choice?  My choice is supported by clinical guidelines  Lack of alternatives in my institution/region  Based on personal experience |
| Q 40 Which of the following strategies do you prefer as second-line treatment for controlling ectopic Cushing’s syndrome?  Bilateral adrenalectomy  Ketoconazole  Metyrapone  Osilodrostat  Combination of ketoconazole/metyrapone and osilodrostat  Somatostatin analogue |
| Q 41 The reasons for switching from first-line to second-line treatment include :  Ineffectiveness  Adverse events  Interaction with other antineoplastic drugs used for the treatment of NETs  Unavailability of first-line drugs in my region  Combination of more than one drug |
| Q 42 If other, please specify |
| Q 43 If you prefer therapy with steroidogenesis inhibitors, which approach do you prefer?  Titration of the drug  Block and replace  Block and replace only in cases of aggressive disease |
| Q 44 If other, please specify |
| Q 45 Which side effect from the list below caused you to discontinue therapy with steroidogenesis inhibitors?  Skin reactions  Hepatotoxicity  Gastrointestinal disorders  None  Other |
| Q 46 If other, please specify |
| Q 47 Does your center use mass spectrometry (MS) to measure 24-hour urinary free cortisol (UFC) during follow-up of medical therapy with Osilodrostat/Metyrapone?  Yes  No  No, but samples can be sent to another center for analysis  No, but samples can be sent to another center for analysis  Other |
| Q 48 If other, please specify |
| Q 49 How long does the laboratory take to provide the result of the 24-hour urinary free cortisol (UFC) measurement by mass spectrometry (MS)?  Less than 7 days  About 7 to 14 days  More than 14 days |
| Q 50 Based on your experience, in what percentage of cases are you able to achieve control of ectopic Cushing’s syndrome with the medical treatments listed above?"  0–20% of cases  20–50% of cases  50–80% of cases  80–100% of cases |
